# Serum creatinine- and cystatin C-based indices are associated with the risk of subsequent sarcopenia: evidence from the China Health and Retirement Longitudinal Study

**DOI:** 10.3389/fnut.2024.1471068

**Published:** 2024-11-20

**Authors:** Xin Ning, Chao Xie, Yaozhong Kong

**Affiliations:** Department of Nephrology, The First People’s Hospital of Foshan, Foshan, China

**Keywords:** creatinine, cystatin C, sarcopenia, skeletal muscle index, risk

## Abstract

**Background:**

Serum creatinine (Cr)- and cystatin C (CysC)-based indices have been suggested as alternative markers for sarcopenia, but their predictive value for sarcopenia risk is uncertain, which was investigated in the present study in the Chinese population with the middle and older ages.

**Methods:**

Data from the China Health and Retirement Longitudinal Study (CHARLS) were collected in the 2011 and 2015 waves. All participants were free of sarcopenia at the baseline. Sarcopenia was diagnosed when low muscle mass and grip strength or low physical performance were present. Four indices were computed: predictive skeletal muscle mass index (pSMI), total-body muscle mass (TBMM), creatinine-to-cystatin C ratio (CCR), and sarcopenia index (SI). Restricted cubic splines and logistic regression models were used to assess the effects of these indices on sarcopenia risk.

**Results:**

Among 4,527 participants without sarcopenia at the baseline (2011), the median age was 58 year-old (IQR: 52–65), with 52.7% women. Followed up in year 2015, the incidence of sarcopenia was 20.8 per 1,000 person-years (376/4,527). Neither CCR nor SI showed linear or non-linear associations with the risk of subsequent sarcopenia. However, a decrease in pSMI and TBMM was significantly associated with an increased risk of sarcopenia [adjusted per-SD decrease OR, 2.93; 95% CI, 2.09–4.13, *p* < 0.001; adjusted per-SD decrease OR: 2.38, 95% CI: 1.80–3.16, *p* < 0.001, respectively].

**Conclusion:**

In the middle and older age of Chinese population, decreased pSMI and TBMM were associated with an increased risk of subsequent sarcopenia, whereas CCR and SI showed no such correlation. Thus, pSMI and TBMM may serve as potential biological indicators for predicting the risk of sarcopenia, and decreased pSMI and TBMM may be the early biomarkers for diagnosis and intervention of sarcopenia.

## 1 Introduction

Sarcopenia is a progressive systemic skeletal muscle disease associated with an increased risk of falls, fractures, disability, and mortality (1, 2). Its specific definition includes low muscle strength, low muscle quantity or quality, and low physical performance (3). Sarcopenia typically affects the middle- and older age of population, as well as individuals with certain chronic conditions (4). A recent meta-analysis indicated that the overall prevalence of sarcopenia ranges from 10 to 27% (5). The early identification of individuals at high risk for potential sarcopenia is a significant challenge, given its significant prevalence and adverse outcomes.

Multiple screening tools that incorporate serum creatinine (Cr) and cystatin C (CysC) levels have been proposed as alternative biological markers for diagnosing sarcopenia (6–9). Serum Cr originates from phosphocreatine and represents the ultimate product of skeletal muscle metabolism; its levels are influenced by kidney function (10). CysC is an ideal endogenous marker that reflects changes in the glomerular filtration rate, and its production is less affected by the muscle mass (6, 11).

The serum Cr-to-cystatin-C ratio (CCR) is the first available index for sarcopenia by estimating the muscle mass and acting as an independent predictor of hospitalization and the 90-day mortality in ICU patients (12). The second index such as the sarcopenia index (SI), defined as serum Cr multiplied by eGFR_CysC_, showed potentially stronger correlations with sarcopenia (6, 13). Kim et al. (14) developed an equation to estimate total-body muscle mass (TBMM) in 2016. Recently, Kusunoki et al. (15) devised an equation for the predictive skeletal muscle mass index (pSMI) among older individuals in Japanese community settings, providing a valuable estimation of muscle mass and diagnosis of sarcopenia. Previous longitudinal studies indicated that lower CCR is associated with higher mortality in patients with acute kidney injury or cancer in community-dwelling populations (16–18).

Given the complexity in diagnosing sarcopenia, several cross-sectional studies have confirmed the high diagnostic value of these indices for sarcopenia or low muscle mass (6, 15, 19–23). However, whether these indices are associated with the risk of sarcopenia remains unknown. Recently, a prospective cohort study suggested an inverse association between CCR and the risk of long-term incidence of self-reported falls among older women in the community (24). Furthermore, there is lack of research investigating the prognostic value of SI, pSMI, and TBMM regarding adverse outcomes clinically. Therefore, in this study, we aimed to estimate the effects of serum Cr- and CysC-based indices for the risk of subsequent development of sarcopenia in the middle and older age of Chinese population and also aimed to identify the early biomarker for diagnosis and intervention of individuals with sarcopenia.

## 2 Materials and methods

### 2.1 Data resource and study population

As previously mentioned, the detailed methodology of the China Health and Retirement Longitudinal Study (CHARLS) has been presented (25, 26). In short, CHARLS is a nationally representative longitudinal survey of the middle-aged and older population in China that was conducted to analyze the issue of population aging in the country. Investigators have conducted nationwide surveys since 2011. All the participants underwent standardized questionnaires, blood tests, and physical examinations every 2–3 years. All participants provided informed consent, and the CHARLS protocol was approved by the Peking University Ethical Review Committee (IRB00001052-11015) following the Declaration of Helsinki.

**Figure 1:**
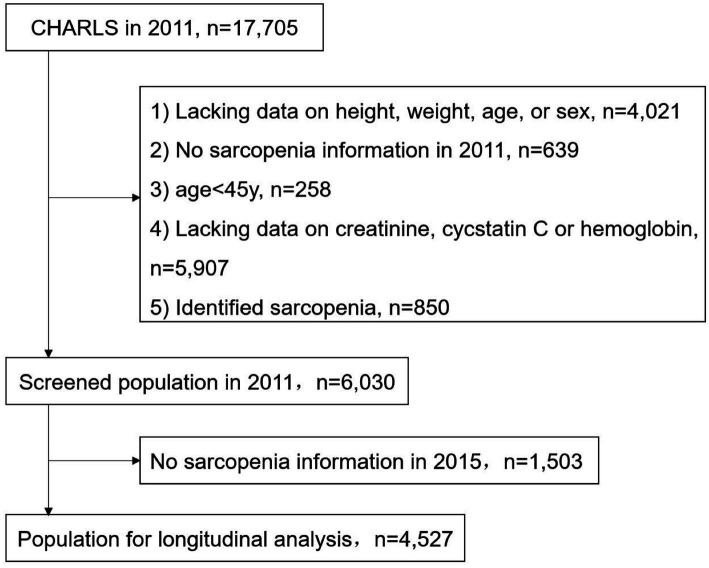
Screening flow chart.

We selected data from the 2011 and 2015 waves, excluding participants with missing baseline data on height, weight, age, and sex (4021); grip strength, walking speed, speed of repeated chair stand, and balance tests (639); serum Cr, CysC, and hemoglobin (5907); age < 45 years (258); and those identified as having sarcopenia at baseline (850) (Figure 1). The identification of baseline sarcopenia is described in the “2.3. Study Outcomes” section of this chapter.

### 2.2 Variables

The blood test measurement methods were according to Zhao et al. (25). The calculation methods for pSMI, TBMM, CCR, and SI are detailed in Supplementary Table S1. eGFR_CysC_ was calculated using the CKD-EPI equation (27). The covariates of interest included age, sex (men or women), place of residence (urban or rural), smoking, drinking, marital status (married or other), educational level (elementary school and below, middle school, high school and above), body mass index (BMI), diastolic blood pressure (DBP), systolic blood pressure (SBP), laboratory test results including triglycerides (TG), low-density lipoprotein cholesterol (LDL-C), C-reactive protein (CRP), glycosylated hemoglobin (HbA1c), hemoglobin, estimated glomerular filtration rate (eGFR), and comorbidities. The comorbidity covariates derived from the questionnaire included self-reported data for 15 diseases: physical disability, hypertension, dyslipidemia, diabetes, cancer, chronic lung disease, liver disease, heart problem, stroke, kidney disease, digestive disease, psychiatric problem, memory-related disease, rheumatism, and asthma. These covariates were obtained through responses to questions such as “Have you been diagnosed with (this) by a doctor?” and “Do you have one of the following disabilities?”.

### 2.3 Study outcomes

Sarcopenia was defined as low muscle mass, grip strength, or physical performance. Having either low grip strength or taking ≥12 s to complete five repetitions of the chair stand test was defined as possible sarcopenia. Sarcopenia was determined based on the consensus of the Asian Working Group for Sarcopenia (AWGS) (1): (1) grip strength (men <28 kg, women <18 kg); (2) physical performance: walking a distance of 2.5 m with a walking speed of <1.0 m/s, taking ≥12 s to complete the 5-time chair stand test, or having a Short Physical Performance Battery (SPPB) score based on the balance ability test of ≤9; (3) muscle mass: estimating the appendicular skeletal muscle mass (ASM) uses an equation validated in the Chinese population, with dual-energy X-ray absorptiometry (DXA) as the gold standard (28, 29). The equation model had a corrected *R*^2^ value of 0.90 (28). The formula for calculating the skeletal muscle index (SMI) was ASM/height^2^, with the unit of height in meters (29). The critical threshold for SMI was set at the lowest 20th percentile within the study population (29). In our analysis, SMI < 6.96 kg/m^2^ for men and SMI < 5.21 kg/m^2^ for women were considered to indicate low muscle mass.

The measurement methods for grip strength and physical performance have been described by Zhao et al. (25). Grip strength was calculated as the average of the maximum readings from two single-handed measurements. The SPPB score was determined based on the duration of the three standing positions (side-by-side, semi-tandem, and full tandem), with scores ranging from 0 to 4 for each position (30). The scores for each position were summed to calculate the total score (30).

### 2.4 Statistical analysis

Categorical variables are presented as absolute numbers and percentages (%). Continuous variables are presented as mean ± standard deviation (SD) for normally distributed data and as median (interquartile range) for non-normally distributed data. For inter-group comparisons, the chi-square test was applied to categorical variables, while the *t*-test or ANOVA was used for continuous variables when normality was met; otherwise, the rank-sum test was employed.

Spearman’s correlation analysis was used to explore the correlation between the four indices and physical examination parameters of age, BMI, and SMI. Logistic regression models were used to estimate the risk of sarcopenia associated with decreases in pSMI, TBMM, CCR, and SI, expressed as odds ratios (ORs) with 95% confidence intervals (CIs). Given that the equations for the four indices involved Cr, cystatin C, age, sex, height, weight, and hemoglobin, we included only the essential confounding variables that we focused on as covariates to avoid multicollinearity. Three adjustment models were used: (1) unadjusted; (2) adjusted for age, sex, and BMI; and (3) further adjusted for region, smoking, marital status, DBP, SBP, TG, LDL-C, HbA1c, disability, heart problem, and kidney- and memory-related disease. The linear trend across quintiles was tested by including quintiles as single continuous variables in logistic regression models. Their non-linear associations were further evaluated using restricted cubic splines in the logistic regression models with five knots at the 5th, 35th, 50th, 65th, and 95th percentiles of the indices (reference value = median). Assuming that the data were missing at random, multiple imputations were performed for missing data.

Subgroup and sensitivity analyses were performed to demonstrate the robustness of the results. Interaction on the multiplicative scale was evaluated using likelihood ratio tests, whereas that on the additive scale was assessed by computing the relative excess risk due to the interaction. In the sensitivity analysis, we reanalyzed possible sarcopenia as an outcome. In an additional analysis, we extracted pSMI, TBMM, CCR, and SI values from 2015, calculated their slopes of change between 2011 and 2015, and analyzed the association between the slopes and risk of sarcopenia, considering the slopes as continuous variables (annual rate of change). All statistical analyses were conducted using R software, version 4.1.2 (R Foundation for Statistical Computing, Vienna, Austria). Statistical significance was set at *p* < 0.05.

## 3 Results

A total of 4,527 participants from the 2011 wave of CHARLS were included in this study (Table 1). Among them, 47.3% were male and 52.8% were female, with a median age of 58 (interquartile range [IQR], 52–65) years. Participants in the pSMI Q1–Q2 group tended to be older, reside in rural areas, and have lower educational levels, and were less likely to be married, smoke, or drink. Notably, participants in the pSMI Q1–Q2 group were more likely to have lower BMI, blood pressure, and blood lipid levels and less likely to have hypertension, diabetes, and dyslipidemia.

**Table 1:** Baseline characteristics of participants.

	Total (*N* = 4,527)	pSMI Q1 (*N* = 1,128)	pSMI Q2 (*N* = 1,136)	pSMI Q3 (*N* = 1,131)	pSMI Q4 (*N* = 1,132)	*P-*value
Women	2,384 (52.7)	1,069 (94.8)	749 (65.9)	428 (37.8)	138 (12.2)	
Age, year	58 (52,65)	61 (55,68)	59 (53,67)	58 (51,65)	56 (50,62)	<0.001
Rural	3,863 (85.4)	1,013 (89.9)	974 (85.7)	987 (87.3)	889 (78.7)	<0.001
Smoke	1753 (38.7)	141 (12.5)	357 (31.5)	566 (50.1)	689 (60.9)	<0.001
Drink	1,143 (25.3)	120 (10.6)	203 (17.9)	336 (29.7)	484 (42.8)	<0.001
Marital status						<0.001
Married	4,033 (89.1)	933 (82.7)	1,005 (88.5)	1,021 (90.3)	1,074 (94.9)	
Other	494 (10.9)	195 (17.3)	131 (11.5)	110 (9.7)	58 (5.1)	
Educational level						<0.001
Elementary school and below	3,199 (70.7)	975 (86.4)	880 (77.5)	769 (68.1)	575 (50.8)	
Middle school	890 (19.7)	105 (9.3)	181 (15.9)	246 (21.8)	358 (31.6)	
High school and above	437 (9.7)	48 (4.3)	75 (6.6)	115 (10.2)	199 (17.6)	
BMI, kg/m^2^	23.5 (21.5,26.1)	22 (20.4,23.5)	23.5 (21.3,25.6)	23.5 (21.3,26.7)	25.7 (23.6,28.1)	<0.001
SBP, mmHg	127.7 (115.3,142.7)	127.3 (113,143.7)	126.7 (114.3,140.7)	126.3 (114.7,142.3)	130.7 (119,144)	<0.001
DBP, mmHg	75 (67.7,83.7)	73 (66.3,81.7)	74 (66.7,82.5)	75.3 (67.4,83.7)	78 (70,86.3)	<0.001
TG, mg/dl	107.1 (77,158.4)	102.7 (76.1,144.3)	103.5 (74.1,148.7)	103.5 (73,154.9)	123 (86.7,187.6)	<0.001
LDL-C, mg/dl	115.2 (93.9,138.4)	118.7 (97.8,141.4)	113.9 (92.4,140.2)	113.5 (91.3,134.8)	114 (94.3,136.9)	<0.001
CRP, mg/l	1 (0.6,2.1)	0.9 (0.5,1.8)	1 (0.6,2)	1.1 (0.5,2.2)	1.2 (0.7,2.4)	<0.001
HbA1c, %	5.1 (4.9,5.4)	5.2 (4.9,5.4)	5.1 (4.9,5.4)	5.1 (4.9,5.4)	5.2 (4.9,5.5)	0.29
Hb, g/dl	14.3 (13.1,15.6)	13.8 (12.7,14.8)	14 (12.9,15.1)	14.4 (13.2,15.8)	15.1 (13.9,16.2)	<0.001
eGFR, mL/min/1.73m^2^	94.8 (84.7,102.4)	93.9 (84,101.8)	94.9 (85.6,102.6)	95 (85,102.4)	95.3 (84.3,102.7)	0.426
Physical disability	148 (3.3)	25 (2.2)	38 (3.4)	35 (3.1)	50 (4.4)	0.032
Hypertension	1,246 (27.7)	251 (22.4)	297 (26.3)	305 (27.1)	393 (35)	0.001
Dyslipidemia	470 (10.6)	85 (7.7)	118 (10.6)	102 (9.2)	165 (14.9)	<0.001
Diabetes	304 (6.8)	59 (5.3)	71 (6.3)	74 (6.6)	100 (8.9)	0.006
Cancer	35 (0.8)	10 (0.9)	9 (0.8)	8 (0.7)	8 (0.7)	0.955
Chronic lung disease	439 (9.7)	105 (9.4)	112 (9.9)	123 (10.9)	99 (8.8)	0.371
Liver disease	144 (3.2)	26 (2.3)	33 (2.9)	35 (3.1)	50 (4.5)	0.032
Heart problem	557 (12.4)	130 (11.6)	134 (11.9)	125 (11.1)	168 (14.9)	0.026
Stroke	87 (1.9)	24 (2.1)	16 (1.4)	21 (1.9)	26 (2.3)	0.439
Kidney disease	272 (6.1)	57 (5.1)	65 (5.8)	74 (6.6)	76 (6.8)	0.313
Digestive disease	994 (22)	286 (25.4)	253 (22.4)	238 (21.1)	217 (19.3)	0.017
Psychiatric problem	60 (1.3)	21 (1.9)	19 (1.7)	9 (0.8)	11 (1)	0.004
Memory-related disease	50 (1.1)	17 (1.5)	13 (1.1)	11 (1)	9 (0.8)	0.416
Rheumatism	1,529 (33.9)	456 (40.5)	395 (34.9)	375 (33.3)	303 (26.8)	<0.001
Asthma	205 (4.5)	55 (4.9)	48 (4.2)	54 (4.8)	48 (4.2)	0.811

Missing data: 5.4%. Data are presented as median (IQR) or *n* (%). BMI, body mass index (calculated as weight in kilograms divided by height in meters squared).SBP, systolic blood pressure; DBP, diastolic blood pressure. TG, triglycerides; LDL-C, low-density lipoprotein cholesterol; CRP, C-reactive protein; HbA1c, glycosylated hemoglobin; Hb, hemoglobin; eGFR, estimated glomerular filtration rate.

During the 4-year follow-up period, there were 376 cases of sarcopenia, with an incidence rate of 20.8 per 1,000 person-years. Spearman’s correlation analysis revealed that pSMI was significantly correlated with SMI (*r* = 0.87, *p* < 0.001) and grip strength (*r* = 0.59, *p* < 0.001). Similarly, TBMM was significantly correlated with SMI (*r* = 0.82, *p* < 0.001) and grip strength (*r* = 0.62, *p* < 0.001). In contrast, CCR and SI exhibited only weak correlations with grip strength (*r* = 0.38, *p* < 0.001; *r* = 0.43, *p* < 0.001) and SMI (*r* = 0.32, *p* < 0.001; *r* = 0.35, *p* < 0.001) (Supplementary Table S2).

Univariate analysis revealed significant associations between all four indices and subsequent sarcopenia (Model 1 in Table 2). However, in multivariable-adjusted logistic regression models, a decrease in pSMI and TBMM were both associated with an increased risk of sarcopenia (adjusted OR: 2.93, 95% CI: 2.09–4.43, *p* < 0.001; adjusted OR: 2.38, 95% CI: 1.80–3.16, *p* < 0.001, respectively), along with their categorical variables. This finding suggests that for each standard deviation (SD) (0.85) decrease in pSMI, the participants experienced a 2.93-fold increase in the risk of developing sarcopenia, whereas for TBMM (6.96), there was a 2.38-fold increase. Participants in the pSMI or TBMM Q1–Q2 groups had a significantly higher risk of sarcopenia than those in the Q4 group (both *P* for trend <0.001). In contrast, CCR and SI were less significant (*P* for trend = 0.044 and 0.054, respectively) (Model 3 in Table 2). Furthermore, using restricted cubic splines, we confirmed that there were no non-linear relationships between these indices and the risk of subsequent sarcopenia (Supplementary Figure S1).

**Table 2:** The effect of pSMI, TBMM, CCR, and SI on the risk of subsequent sarcopenia.

	No of events/No of total population	Incidence rate per 1,000 person years	Model 1		Model 2		Model 3		*P* for trend
			OR (95%CI)	*P-*value	OR (95%CI)	*P-*value	OR (95%CI)	*P-*value	
pSMI per-SD decrease	376/4,527	20.8	3.23 (2.80–3.75)	<0.001	3.21 (2.31–4.47)	<0.001	2.93 (2.09–4.13)	<0.001	
Q1	243/1,132	53.7	24.62 (13.69–49.99)	<0.001	8.05 (3.56–19.58)	<0.001	6.40 (2.79–15.79)	<0.001	
Q2	105/1,132	23.2	11.79 (6.45–24.17)	<0.001	3.20 (1.63–6.90)	0.001	2.68 (1.36–5.83)	0.007	
Q3	22/1,129	4.9	5.74 (3.04–12.00)	<0.001	1.84 (0.94–3.96)	0.091	1.64 (0.84–3.54)	0.174	
Q4	6/1,134	1.3	Ref	NA	Ref	NA	Ref	NA	<0.001
TBMM per-SD decrease	376/4,527	20.8	2.56 (2.23–2.95)	<0.001	2.56 (1.95–3.37)	<0.001	2.38 (1.80–3.16)	<0.001	
Q1	188/1,132	41.5	17.14 (10.11–31.82)	<0.001	8.67 (4.19–18.95)	<0.001	7.00 (3.33–15.50)	<0.001	
Q2	84/1,131	18.6	6.91 (3.97–13.05)	<0.001	3.30 (1.77–6.57)	<0.001	2.87 (1.52–5.76)	0.002	
Q3	91/1,132	20.1	7.52 (4.34–14.18)	<0.001	2.65 (1.49–5.13)	0.002	2.37 (1.31–4.60)	0.041	
Q4	13/1,132	2.9	Ref	NA	Ref	NA	Ref	NA	<0.001
CCR per-SD decrease	376/4,527	20.8	1.59 (1.40–1.82)	<0.001	1.28 (1.10–1.49)	0.002	1.22 (1.05–1.44)	0.013	
Q1	243/1,132	53.7	2.83 (2.05–3.98)	<0.001	1.63 (1.11–2.43)	0.014	1.43 (0.96–2.15)	0.079	
Q2	105/1,132	23.2	2.31 (1.65–3.26)	<0.001	1.66 (1.13–2.45)	0.010	1.49 (1.01–2.21)	0.048	
Q3	22/1,129	4.9	1.47 (1.03–2.13)	0.037	1.23 (0.82–1.84)	0.319	1.15 (0.76–1.73)	0.514	
Q4	6/1,134	1.3	Ref	NA	Ref	NA	Ref	NA	0.044
SI per-SD decrease	376/4,527	20.8	1.79 (1.58–2.03)	<0.001	1.25 (1.07–1.47)	0.005	1.20 (1.02–1.41)	0.029	
Q1	137/1,132	30.3	3.92 (2.79–5.63)	<0.001	1.61 (1.06–2.48)	0.027	1.44 (0.94–2.23)	0.099	
Q2	101/1,129	22.4	3.00 (2.11–4.34)	<0.001	1.73 (1.16–2.62)	0.008	1.57 (1.05–2.40)	0.031	
Q3	73/1,134	16.1	1.44 (0.97–2.16)	0.073	1.19 (0.77–1.85)	0.435	1.13 (0.73–1.76)	0.585	
Q4	65/1,132	14.4	Ref	NA	Ref	NA	Ref	NA	0.055

Model 1: unadjusted; Model 2: adjusted for age, sex, BMI; Model 3: further adjusted for region, smoke, marital status, DBP, SBP, TG, LDL-C, HbA1c, disability, heart problem, kidney and memory-related disease. *P* for trend was computed in Model 3. pSMI, predictive skeletal muscle mass index; TBMM, estimate total-body muscle mass; CCR, creatinine-to-cystatin C ratio; SI, sarcopenia index.

**Figure 2:**
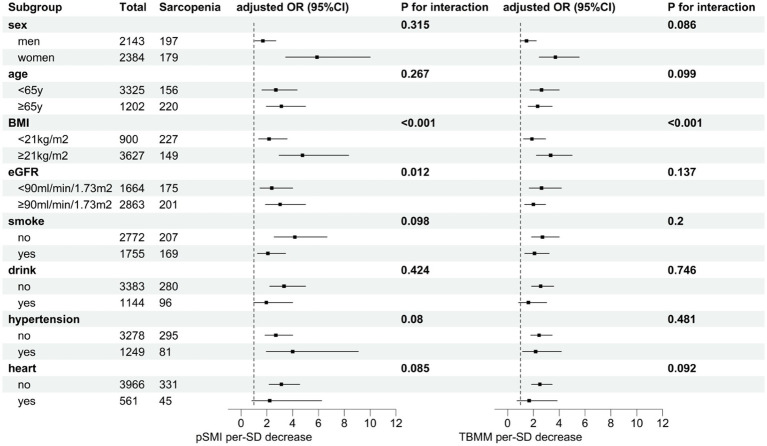
Subgroup analysis of the effect of a one-standard-deviation decrease in pSMI or TBMM on the risk of subsequent sarcopenia. Adjusted for the region, smoking, marital status, DBP, SBP, TG, LDL-C, HbA1c, disability, heart problem, kidney, and memory-related disease.

Subgroup analysis revealed that the association between pSMI or TBMM and subsequent sarcopenia risk was consistent across most subgroups. However, the impact of decreased pSMI on sarcopenia risk varied significantly among participants with different BMI and eGFR levels. Specifically, the effect of decreased pSMI on sarcopenia risk was more pronounced in participants with higher BMI (BMI ≥ 21 kg/m^2^) (*P* for interaction <0.001) and higher eGFR (eGFR ≥90 mL/min/1.73 m^2^) (*P* for interaction = 0.012). Similarly, the effect of decreased TBMM on sarcopenia risk was more evident in participants with higher BMI (BMI ≥ 21 kg/m^2^) (*P* for interaction <0.001) (Figure 2; Supplementary Table S3). Conversely, CCR and SI did not exhibit consistent trends in the subgroup analysis (Supplementary Figure S2 and Supplementary Table S4). Interestingly, when we changed the outcome to possible sarcopenia, we observed that decreases in all four indices were associated with an increased risk of subsequent possible sarcopenia (Supplementary Table S5).

Similar results were found in our additional analysis, where the slopes of pSMI and TBMM changes were also associated with the risk of sarcopenia (adjusted OR: 2.20; 95% CI: 1.83–2.64, *p* < 0.001; adjusted OR: 1.70; 95% CI: 1.38–2.11, *p* < 0.001, respectively), while the slopes of CCR and SI changes were not significant (Table 3). This finding means that for individuals, a decrease of 1 SD per year (1.48%/year) in pSMI and 1 SD per year (3.49%/year) in TBMM increased the risk of sarcopenia by 2.03- and 1.58-fold, respectively.

**Table 3:** The effect of the slopes of pSMI, TBMM, CCR, and SI changes on the risk of subsequent sarcopenia.

	Model 1		Model 2		Model 3		*P* for trend
	OR (95%CI)	*P-*value	OR (95%CI)	*P-*value	OR (95%CI)	*P-*value	
pSMI slope per-SD decrease, %	1.25 (1.10–1.42)	0.001	1.91 (1.62–2.26)	<0.001	2.03 (1.71–2.41)	<0.001	
Q1	1.53 (1.10–2.15)	0.012	4.36 (2.89–6.64)	<0.001	4.81 (3.16–7.40)	<0.001	
Q2	1.17 (0.83–1.67)	0.371	2.49 (1.64–3.82)	<0.001	2.64 (1.72–4.01)	<0.001	
Q3	1.07 (0.75–1.53)	0.714	2.02 (1.33–3.10)	0.001	2.11 (1.38–3.25)	<0.001	
Q4	Ref	NA	Ref	NA	Ref	NA	<0.001
TBMM slope per-SD decrease, %	1.09 (0.96–1.24)	0.175	1.47 (1.21–1.79)	<0.001	1.58 (1.29–1.95)	<0.001	
Q1	1.41 (1.00–2.00)	0.055	3.51 (2.16–5.77)	<0.001	4.17 (2.51–7.01)	<0.001	
Q2	1.35 (0.95–1.92)	0.093	2.37 (1.50–3.76)	<0.001	2.63 (1.64–4.24)	<0.001	
Q3	1.28 (0.90–1.82)	0.179	1.71 (1.12–2.62)	0.014	1.86 (1.21–2.88)	0.005	
Q4	Ref	NA	Ref	NA	Ref	NA	<0.001
CCR slope per-SD decrease, %	0.87 (0.79–0.96)	0.003	0.86 (0.76–0.99)	0.023	0.88 (0.77–1.02)	0.077	
Q1	0.61 (0.43–0.85)	0.004	0.85 (0.57–1.25)	0.403	0.95 (0.63–1.42)	0.791	
Q2	0.62 (0.44–0.87)	0.006	0.82 (0.56–1.21)	0.332	0.88 (0.59–1.30)	0.524	
Q3	0.85 (0.62–1.16)	0.301	0.86 (0.59–1.24)	0.408	0.88 (0.60–1.27)	0.48	
Q4	Ref	NA	Ref	NA	Ref	NA	0.740
SI slope per-SD decrease, %	0.86 (0.78–0.94)	0.001	0.87 (0.78–0.97)	0.008	0.88 (0.79–0.99)	0.017	
Q1	0.62 (0.44–0.86)	0.005	0.95 (0.64–1.39)	0.786	1.04 (0.69–1.54)	0.864	
Q2	0.54 (0.38–0.77)	<0.001	0.75 (0.50–1.11)	0.153	0.81 (0.54–1.21)	0.310	
Q3	0.85 (0.62–1.16)	0.306	1.03 (0.72–1.48)	0.876	1.07 (0.74–1.54)	0.724	
Q4	Ref	NA	Ref	NA	Ref	NA	0.778

Model 1: unadjusted; Model 2: adjusted for age, sex, BMI; Model 3: further adjusted for region, smoke, marital status, DBP, SBP, TG, LDL-C, HbA1c, disability, heart problem, kidney and memory-related disease. *P* for trend was computed in Model 3. pSMI, predictive skeletal muscle mass index; TBMM, estimate total-body muscle mass; CCR, creatinine-to-cystatin C ratio; SI, sarcopenia index.

## 4 Discussion

In this longitudinal study with the middle and older age of population (aged ≥45 years) in China, we found that among the four indices based on serum Cr and cystatin C, only the decrease in pSMI and TBMM was associated with an increased risk of the subsequent development of sarcopenia. In contrast, the previously recognized CCR and SI indexes were not. This finding suggests that pSMI and TBMM may act as biomarkers for predicting the risk of sarcopenia in the middle and older age of Chinese populations. Decreased pSMI and TBMM may be the early indicators for both diagnosis and intervention of patients with sarcopenia.

It has been reported that the CCR and SI are biomarkers for sarcopenia screening in populations with cancer or other specific diseases, as well as among community-dwelling individuals (6, 21–24). Recent studies also found pSMI and TBMM [area under the receiver operating characteristic curves (AUC): 0.68–0.77] produces a better diagnostic value than CCR and SI (AUC: 0.57–0.65) for patients with sarcopenia in community-dwelling individuals aged ≥65 years at the cross-sectional level (19, 20). Similarly, Kitago et al. (21) reported no significant dose–response relationship between CCR Q1–Q4 and changes in sarcopenia prevalence, SMI, grip strength, and walking speed over the subsequent 6 years (21). In consistent with these findings, we also found that there was neither a significant linear nor a non-linear relationship between CCR or SI and the risk of sarcopenia, suggesting that CCR and SI may not be useful predictors of sarcopenia. In addition, CCR and SI also showed weaker correlations with grip strength and SMI but not with BMI. The advantage of pSMI and TBMM lies in their incorporation of factors, such as body weight, height, age, and sex, making them more comprehensive indices. They are also closely related to the development of grip strength, SMI, and BMI.

There was significant heterogeneity in the relationships between the four indices and the outcomes at different BMI levels. BMI reflects both lean body mass and fat mass, and individuals with varying BMI may exhibit differences in the distribution of these components (31). The predicted proportion of lean body mass decreased with higher BMI (31). Our findings suggest the interaction between BMI, pSMI, and TBMM, indicating the need for further studies to explore this relationship. A previous study did not consistently associate CCR with muscle mass in community-dwelling older adults (32). However, owing to the small sample size of urban participants, we did not extract urban areas as a subgroup. Additionally, CCR and SI were not associated with sarcopenia in almost all subgroups in our analysis.

The primary advantage is our thorough evaluation of the impact of serum Cr- and cystatin C-based measures on subsequent sarcopenia among middle-aged and older Chinese adults in a nationally representative retrospective cohort study. Our study had some limitations. Sarcopenia was defined based on parameters such as grip strength, walking speed, and the ASM equation, without using DXA or bioelectrical impedance analysis. This limitation could potentially affect the accuracy of the definition of sarcopenia; however, conducting the above tests on a large sample size might be impractical. Furthermore, it is worth noting that the ASM equation used was previously validated in the Chinese population (28). Second, this was a retrospective study. Although we had made the justification for several potential confounders in our analysis, other confounding factors may remain and should be considered, such as the serum albumin level for reflecting the nutritional status; the physical activity which is crucial for muscle mass; and thyroid malfunction and corticosteroid therapy, which may affect the serum CysC levels. Third, we did not consider the timescale in our additional analysis. Notably, the indices may change over time with a fixed effect, which could affect the interpretation of our findings. Future research could benefit from incorporating time-dependent analyses to better understand the evolution of these indices and their implications for sarcopenia risk assessment. Fourth, although we adopted a stringent identification process for sarcopenia, some cases may not have been captured, potentially introducing bias into the incidence calculation. Nevertheless, our findings suggest that the incidence rate falls within a reasonable range compared to other studies (33, 34). Finally, our study was conducted in a community population aged ≥45 years in China, and the findings may not be generalizable to other populations with specific diseases, other ethnic groups, or populations with different lifestyles or healthcare systems.

## 5 Conclusion

Our findings underscore the value of pSMI and TBMM as risk factors and reliable predictors for sarcopenia in middle and older age of Chinese population. Conversely, CCR and SI may not offer the same level of effectiveness. The use of straightforward equations to calculate pSMI and TBMM may enable the early identification of individuals with a high risk of sarcopenia, which may offer timely intervention and management clinically. However, further research is required to validate these findings in a larger population.

## Data Availability

The raw data supporting the conclusions of this article will be made available by the authors, without undue reservation.
