# Supplementary material for: Serum creatinine- and cystatin C-based indices are associated with the risk of subsequent sarcopenia: evidence from the China Health and Retirement Longitudinal Study
Source: Front Nutr. 2024 Nov 20;11:1471068. doi: 10.3389/fnut.2024.1471068 (PMC11614666; doi:10.3389/fnut.2024.1471068)
Supplement: Supplementary file 1 [file Data_Sheet_1.docx]

Table S1. The calculation equation of the indices.

|  | equation | reference |
| --- | --- | --- |
| CCR | Cr / CysC × 100 | [4] |
| SI | Cr × eGFR_CysC_ | [4,11] |
| eGFR_CysC_ | CysC ≤ 0.8mg/l: 133 × (CysC / 0.8)^-0.499^ × 0.996^age^ [×0.932 if female]  CysC > 0.8mg/l: 133 × (CysC / 0.8)^-1.328^ × 0.996^age^ [×0.932 if female] | [25] |
| pSMI | men: 4.17 – 0.012 × age + 1.24 × (Cr / CysC) – 0.0513 × Hb + 0.0598 × body weight  women: 3.55 –0.00765 × age + 0.852 × (Cr / CysC) – 0.0627 × Hb + 0.0614 × body weight | [13] |
| TBMM | men: Cr × body weight / (0.00675 × body weight × CysC + Cr)  women: Cr × body weight / (0.01006 × body weight × CysC + Cr) | [12] |
| ASM | 0.193 × body weight + 0.107 × height – 4.157 × sex – 0.037 × age – 2.631 | [26,27] |

Cr represents creatinine (mg/dl), CysC represents Cystatin C (mg/l), and Hb represents hemoglobin (g/dl). Other units include body weight (kg), height (cm), and age (years). Sex was coded as 1 for men and 2 for women. ASM was calculated to identify sarcopenia.

Abbreviations: CCR, creatinine-to-cystatin C ratio; SI, sarcopenia index; pSMI, predictive skeletal muscle mass index; TBMM, estimate total-body muscle mass; ASM, appendicular skeletal muscle mass; eGFR, estimated glomerular filtration rate.

Table S2. Pearson’s correlation analysis of the association between indices (pSMI, TBMM, CCR and SI) and physical examination parameters, age, and BMI.

| variables | pSMI | | TBMM | | CCR | | SI | |
| --- | --- | --- | --- | --- | --- | --- | --- | --- |
|  | r | *P* | r | *P* | r | *P* | r | *P* |
| Age, years | -0.13 | <0.001 | -0.07 | <0.001 | -0.12 | <0.001 | -0.26 | <0.001 |
| BMI, kg/m^2^ | 0.48 | <0.001 | 0.32 | <0.001 | 0.00 | 0.813 | -0.02 | 0.418 |
| 2.5 meters walking speed, m/s | 0.17 | <0.001 | 0.19 | <0.001 | 0.15 | <0.001 | 0.22 | <0.001 |
| 5-time chair stand test time, s | -0.04 | 0.054 | -0.04 | 0.061 | -0.02 | 0.201 | -0.03 | 0.119 |
| SPPB score | 0.06 | 0.002 | 0.07 | <0.001 | 0.07 | <0.001 | 0.11 | <0.001 |
| Grip strength, kg | 0.58 | <0.001 | 0.60 | <0.001 | 0.31 | <0.001 | 0.40 | <0.001 |
| SMI | 0.86 | <0.001 | 0.81 | <0.001 | 0.26 | <0.001 | 0.34 | <0.001 |

Abbreviations: pSMI, predictive skeletal muscle mass index; TBMM, estimate total-body muscle mass; CCR, creatinine-to-cystatin C ratio; SI, sarcopenia index; SPPB, Short Physical Performance Battery; BMI, body mass index, SMI, skeletal muscle index.


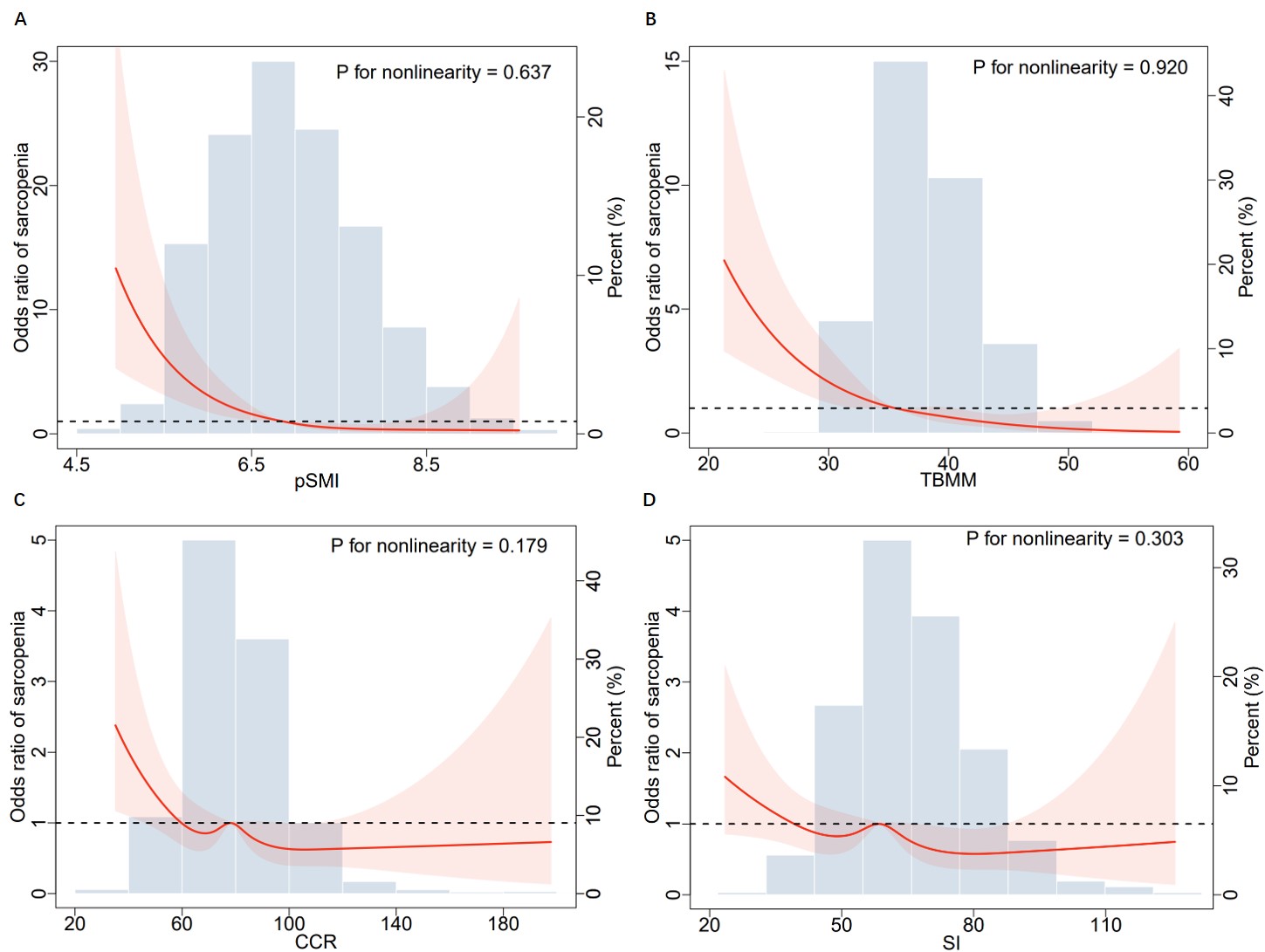


Figure S1. Restricted cubic spline plots for sarcopenia by pSMI, TBMM, CCR and SI levels after covariate adjustment. The background histograms (light blue colour) represent the percent of the density distribution of pSMI, TBMM, CCR and SI in the study population (right y-axis). Heavy central lines represent the estimated adjusted odds ratios, with shaded ribbons denoting 95% confidence intervals. The horizontal dotted lines represent the odds ratio of 1.0.

(A) Restricted cubic spline plot by pSMI. (B) Restricted cubic spline plot by TBMM. (C)Restricted cubic spline plot by CCR. (D) Restricted cubic spline plot by SI.

Abbreviations: pSMI, predictive skeletal muscle mass index; TBMM, estimate total-body muscle mass; CCR, creatinine-to-cystatin C ratio; SI, sarcopenia index.

Table S3. Subgroup analysis of the effect of a one-standard-deviation decrease in pSMI or TBMM on the risk of subsequent sarcopenia.

|  |  |  | pSMI | | | TBMM | | |
| --- | --- | --- | --- | --- | --- | --- | --- | --- |
| Subgroup | Total | Event | OR (95%CI) | *P* value | *P* for interaction | OR (95%CI) | *P* value | *P* for interaction |
| gender |  |  |  |  | 0.315 |  |  | 0.086 |
| men | 2143 | 197 | 1.69 (1.06-2.70) | 0.026 |  | 1.47 (0.98-2.22) | 0.064 |  |
| women | 2384 | 179 | 5.88 (3.45-10.00) | <0.001 |  | 3.70 (2.44-5.56) | <0.001 |  |
| age |  |  |  |  | 0.267 |  |  | 0.099 |
| < 65y | 3325 | 156 | 2.70 (1.61-4.35) | <0.001 |  | 2.63 (1.72-4.00) | <0.001 |  |
| ≥ 65y | 1202 | 220 | 3.13 (1.96-5.00) | <0.001 |  | 2.33 (1.59-3.45) | <0.001 |  |
| BMI |  |  |  |  | <0.001 |  |  | <0.001 |
| < 21kg/m^2^ | 900 | 227 | 2.17 (1.35-3.57) | 0.002 |  | 1.89 (1.22-2.94) | 0.004 |  |
| ≥ 21kg/m^2^ | 3627 | 149 | 4.76 (2.94-8.33) | <0.001 |  | 3.33 (2.22-5.00) | <0.001 |  |
| eGFR |  |  |  |  | 0.012 |  |  | 0.137 |
| < 90ml/min/1.73m^2^ | 1664 | 175 | 2.38 (1.47-4.00) | 0.001 |  | 2.63 (1.67-4.17) | <0.001 |  |
| ≥ 90ml/min/1.73m^2^ | 2863 | 201 | 3.03 (1.89-5.00) | <0.001 |  | 2.00 (1.32-2.94) | 0.001 |  |
| smoke |  |  |  |  | 0.098 |  |  | 0.2 |
| no | 2771 | 207 | 4.17 (2.56-6.67) | <0.001 |  | 2.70 (1.85-4.00) | <0.001 |  |
| yes | 1756 | 169 | 2.08 (1.27-3.45) | 0.004 |  | 2.08 (1.33-3.23) | 0.001 |  |
| drink |  |  |  |  | 0.424 |  |  | 0.746 |
| no | 3383 | 280 | 3.33 (2.22-5.00) | <0.001 |  | 2.56 (1.85-3.57) | <0.001 |  |
| yes | 1144 | 96 | 1.96 (0.98-4.00) | 0.06 |  | 1.61 (0.85-3.03) | 0.141 |  |
| hypertension |  |  |  |  | 0.08 |  |  | 0.481 |
| no | 3276 | 295 | 2.70 (1.85-4.00) | <0.001 |  | 2.44 (1.79-3.45) | <0.001 |  |
| yes | 1251 | 81 | 4.00 (1.96-9.09) | 0.001 |  | 2.17 (1.15-4.17) | 0.019 |  |
| heart problem |  |  |  |  | 0.085 |  |  | 0.092 |
| no | 3966 | 331 | 3.13 (2.17-4.55) | <0.001 |  | 2.50 (1.85-3.45) | <0.001 |  |
| yes | 561 | 45 | 2.22 (0.84-6.25) | 0.117 |  | 1.67 (0.72-3.85) | 0.227 |  |

Adjusted for region, smoke, marital status, DBP, SBP, TG, LDL-C, HbA1c, disability, heart problem, kidney and memory-related disease.

Abbreviations: pSMI, predictive skeletal muscle mass index; TBMM, estimate total-body muscle mass; eGFR, estimated glomerular filtration rate; BMI, body mass index.

Table S4. Subgroup analysis of the effect of a one-standard-deviation decrease in CCR or SI on the risk of subsequent sarcopenia.

|  |  |  | CCR | | | SI | | |
| --- | --- | --- | --- | --- | --- | --- | --- | --- |
| Subgroup | Total | Event | OR (95%CI) | *P* value | *P* for interaction | OR (95%CI) | *P* value | *P* for interaction |
| gender |  |  |  |  | 0.077 |  |  | 0.152 |
| men | 2143 | 197 | 1.02 (0.82-1.28) | 0.889 |  | 1.02 (0.82-1.28) | 0.828 |  |
| women | 2384 | 179 | 1.45 (1.15-1.85) | 0.002 |  | 1.43 (1.11-1.85) | 0.006 |  |
| age |  |  |  |  | 0.126 |  |  | 0.08 |
| < 65y | 3325 | 156 | 1.37 (1.08-1.75) | 0.013 |  | 1.32 (1.04-1.69) | 0.025 |  |
| ≥ 65y | 1202 | 220 | 1.18 (0.96-1.45) | 0.13 |  | 1.16 (0.93-1.45) | 0.184 |  |
| BMI |  |  |  |  | 0.804 |  |  | 0.274 |
| < 21kg/m^2^ | 900 | 227 | 1.28 (1.03-1.61) | 0.027 |  | 1.22 (0.98-1.52) | 0.078 |  |
| ≥ 21kg/m^2^ | 3627 | 149 | 1.20 (0.96-1.52) | 0.114 |  | 1.23 (0.97-1.59) | 0.097 |  |
| eGFR |  |  |  |  | 0.385 |  |  | 0.823 |
| < 90ml/min/1.73m^2^ | 1664 | 175 | 1.37 (1.08-1.75) | 0.014 |  | 1.32 (1.04-1.69) | 0.023 |  |
| ≥ 90ml/min/1.73m^2^ | 2863 | 201 | 0.92 (0.73-1.16) | 0.455 |  | 0.94 (0.74-1.20) | 0.665 |  |
| smoke |  |  |  |  | 0.578 |  |  | 0.997 |
| no | 2771 | 207 | 1.18 (0.97-1.45) | 0.099 |  | 1.20 (0.97-1.49) | 0.093 |  |
| yes | 1756 | 169 | 1.28 (0.99-1.69) | 0.063 |  | 1.19 (0.93-1.54) | 0.181 |  |
| drink |  |  |  |  | 0.549 |  |  | 0.563 |
| no | 3383 | 280 | 1.27 (1.06-1.54) | <0.001 |  | 1.25 (1.03-1.52) | 0.023 |  |
| yes | 1144 | 96 | 1.08 (0.79-1.52) | 0.06 |  | 1.05 (0.76-1.47) | 0.747 |  |
| hypertension |  |  |  |  | 0.391 |  |  | 0.308 |
| no | 3276 | 295 | 1.28 (1.08-1.54) | 0.006 |  | 1.28 (1.08-1.54) | 0.007 |  |
| yes | 1251 | 81 | 1.00 (0.70-1.43) | 0.98 |  | 0.91 (0.63-1.33) | 0.621 |  |
| heart problem |  |  |  |  | 0.015 |  |  | 0.011 |
| no | 3966 | 331 | 1.30 (1.10-1.54) | 0.003 |  | 1.27 (1.06-1.52) | 0.008 |  |
| yes | 561 | 45 | 0.88 (0.62-1.30) | 0.496 |  | 0.82 (0.53-1.28) | 0.364 |  |

Adjusted for region, smoke, marital status, DBP, SBP, TG, LDL-C, HbA1c, disability, heart problem, kidney and memory-related disease.

Abbreviations: CCR, creatinine-to-cystatin C ratio; SI, sarcopenia index; eGFR, estimated glomerular filtration rate; BMI, body mass index.


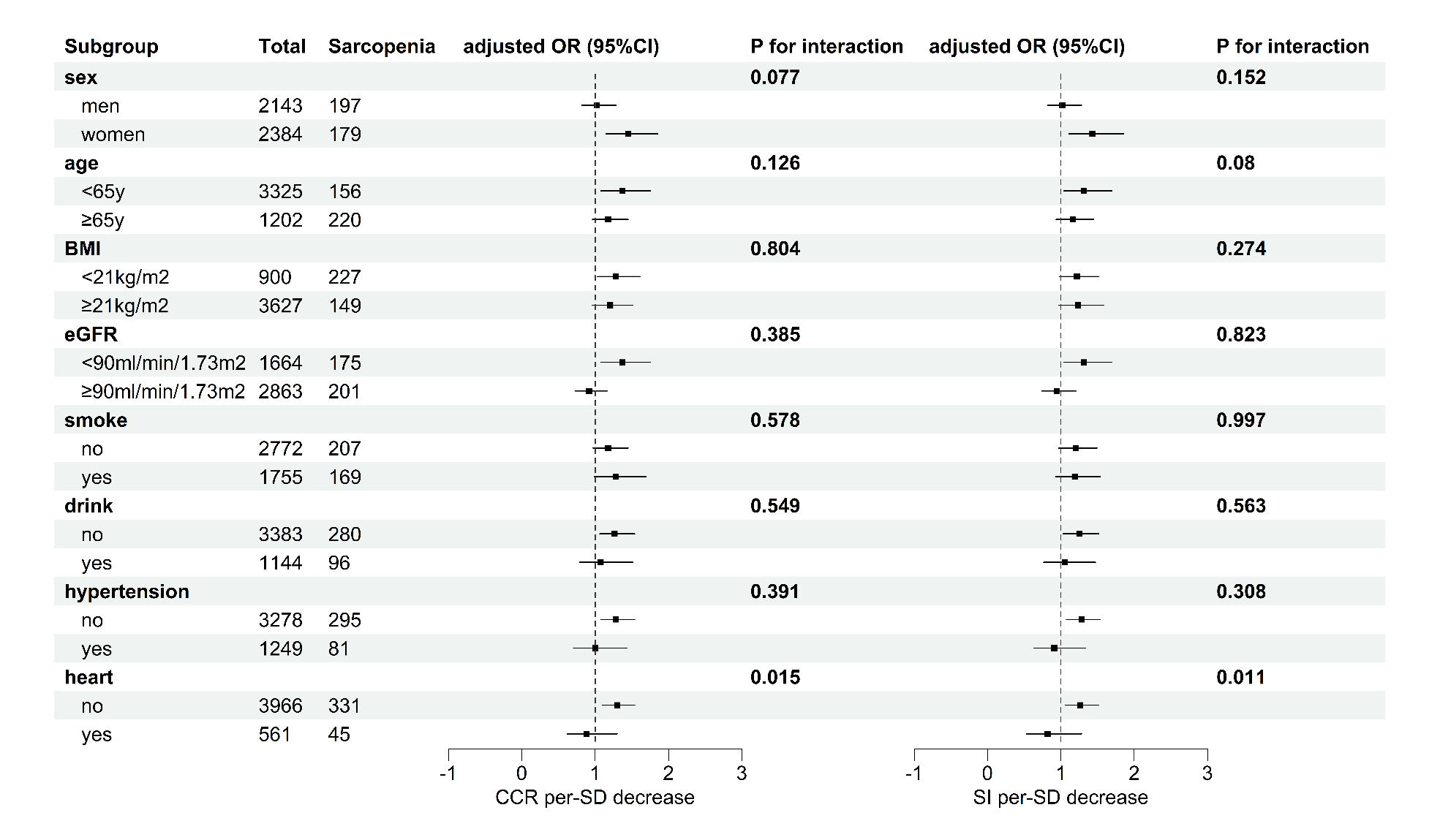


Figure S2. Subgroup analysis of the effect of a one-standard-deviation decrease in CCR or SI on the risk of subsequent sarcopenia.

Adjusted for region, smoke, marital status, DBP, SBP, TG, LDL-C, HbA1c, disability, heart problem, kidney and memory-related disease.

Abbreviations: CCR, creatinine-to-cystatin C ratio; SI, sarcopenia index; eGFR, estimated glomerular filtration rate; BMI, body mass index.

Table S5. The effect of pSMI, TBMM, CCR, and SI on the risk of subsequent possible sarcopenia.

|  | No of events / No of total population | Incidence rate per 1000 person years | Model 1 | | Model 2 | | Model 3 | | |
| --- | --- | --- | --- | --- | --- | --- | --- | --- | --- |
|  |  |  | OR (95%CI) | *P* value | OR (95%CI) | *P* value | OR (95%CI) | *P* value | *P* for trend |
| pSMI per-SD decrease | 1406 / 4527 | 77.6 | 1.32 (1.24-1.41) | <0.001 | 1.85 (1.57-2.17) | <0.001 | 1.74 (1.48-2.06) | <0.001 |  |
| Q1 | 432 / 1132 | 95.4 | 1.90 (1.58-2.28) | <0.001 | 2.35 (1.64-3.38) | <0.001 | 2.09 (1.45-3.02) | <0.001 |  |
| Q2 | 343 / 1132 | 75.8 | 1.79 (1.49-2.15) | <0.001 | 1.99 (1.51-2.62) | <0.001 | 1.84 (1.39-2.42) | <0.001 |  |
| Q3 | 326 / 1129 | 72.2 | 1.32 (1.09-1.59) | 0.004 | 1.37 (1.09-1.72) | 0.007 | 1.29 (1.02-1.62) | 0.031 |  |
| Q4 | 305 / 1134 | 67.2 | Ref | NA | Ref | NA | Ref | NA | <0.001 |
| TBMM per-SD decrease | 1406 / 4527 | 77.6 | 1.30 (1.22-1.39) | <0.001 | 1.70 (1.49-1.93) | <0.001 | 1.62 (1.42-1.85) | <0.001 |  |
| Q1 | 432 / 1132 | 95.4 | 1.94 (1.62-2.33) | <0.001 | 3.05 (2.20-4.23) | <0.001 | 2.72 (1.95-3.80) | <0.001 |  |
| Q2 | 343 / 1132 | 75.8 | 1.44 (1.20-1.74) | <0.001 | 2.04 (1.56-2.67) | <0.001 | 1.90 (1.45-2.50) | <0.001 |  |
| Q3 | 326 / 1129 | 72.2 | 1.44 (1.20-1.73) | <0.001 | 1.63 (1.32-2.03) | <0.001 | 1.53 (1.23-1.91) | <0.001 |  |
| Q4 | 305 / 1134 | 67.2 | Ref | NA | Ref | NA | Ref | NA | <0.001 |
| CCR per-SD decrease | 1406 / 4527 | 77.6 | 1.41 (1.31-1.52) | <0.001 | 1.26 (1.17-1.36) | <0.001 | 1.23 (1.14-1.34) | <0.001 |  |
| Q1 | 442 / 1132 | 97.6 | 2.41 (2.00-2.89) | <0.001 | 1.89 (1.54-2.32) | <0.001 | 1.80 (1.46-2.22) | <0.001 |  |
| Q2 | 370 / 1129 | 81.9 | 1.88 (1.56-2.26) | <0.001 | 1.62 (1.32-1.98) | <0.001 | 1.55 (1.26-1.90) | <0.001 |  |
| Q3 | 314 / 1134 | 69.2 | 1.33 (1.09-1.61) | 0.004 | 1.23 (1.00-1.50) | 0.046 | 1.20 (0.98-1.47) | 0.079 |  |
| Q4 | 280 / 1132 | 61.8 | Ref | NA | Ref | NA | Ref | NA | <0.001 |
| SI per-SD decrease | 1406 / 4527 | 77.6 | 1.57 (1.47-1.69) | <0.001 | 1.29 (1.0.18-1.40) | <0.001 | 1.26 (1.16-1.37) | <0.001 |  |
| Q1 | 444 / 1134 | 97.9 | 3.02 (2.51-3.65) | <0.001 | 1.84 (1.48-2.29) | <0.001 | 1.76 (1.41-2.20) | <0.001 |  |
| Q2 | 380 / 1129 | 84.1 | 2.09 (1.73-2.53) | <0.001 | 1.60 (1.31-1.97) | <0.001 | 1.54 (1.25-1.91) | <0.001 |  |
| Q3 | 307 / 1132 | 67.8 | 1.34 (1.10-1.64) | 0.003 | 1.22 (0.99-1.50) | 0.057 | 1.22 (0.99-1.50) | 0.066 |  |
| Q4 | 275 / 1132 | 60.7 | Ref | NA | Ref | NA | Ref | NA | <0.001 |

Model 1: unadjusted; Model 2: adjusted for age, sex, BMI; Model 3: further adjusted for region, smoke, marital status, DBP, SBP, TG, LDL-C, HbA1c, disability, heart problem, kidney and memory-related disease. *P* for trend was computed in Model 3.

Abbreviations: pSMI, predictive skeletal muscle mass index; TBMM, estimate total-body muscle mass; CCR, creatinine-to-cystatin C ratio; SI, sarcopenia index
